# First description of the female of *Sinopodayichangensis* Zhu, Zhong & Yang, 2020 (Araneae, Sparassidae)

**DOI:** 10.3897/zookeys.1067.72419

**Published:** 2021-10-29

**Authors:** Li-Jun Gong, Yang Zhong

**Affiliations:** 1 Hubei Key Laboratory of Radiation Chemistry and Functional Materials, School of Nuclear Technology and Chemistry & Biology, Hubei University of Science and Technology, Xianning 437100, Hubei, China Hubei University of Science and Technology Xianning China

**Keywords:** Biodiversity, huntsman spiders, taxonomy, Yichang

## Abstract

*Sinopodayichangensis* Zhu, Zhong & Yang, 2020 was described from a single male from Qiaoliao Village, Hubei Province, China. To date, no additional specimens have been recorded. The female is reported for the first time from the type locality. Detailed morphological descriptions of the female, with photographs of living specimens and copulatory organs, are provided.

## Introduction

The genus *Sinopoda* Jäger, 1999 is the fourth largest genus of the family Sparassidae, with 133 species (WSC 2021). To date, *Sinopoda* is distributed from high to low altitude in south, east, and southeast Asia, frequently co-distributed, some of which are located in caves. More than half of the known species were described based on a single sex in *Sinopoda*. From China, 71 species are known; among them, 19 species are only known from females and five only from males (WSC 2021). *Sinopodayichangensis* Zhu, Zhong & Yang, 2020 was described based on a single male specimen from Qiaoliao Village of Hubei Province, China (Zhu et al. 2020). Recently, new material containing both sexes were collected from the type locality. In addition, based on the similar body coloration patterns, we were able to match the females and males together.

## Materials and methods

Specimens were examined and measured with a Leica M205C stereomicroscope. The points arising from the tegular appendages are listed as clock positions from the left bulb in ventral view. Male and female copulatory organs were examined and illustrated after dissection from the spider bodies; vulvae were cleared with Proteinase K. All photographs were taken with a Leica DFC450 digital camera attached to a Leica M205C stereomicroscope, with 10–20 photographs taken in different focal planes and combined using the image stacking software Leica LAS V4.8. Images were edited using Adobe Photoshop CC 2018.

Leg measurements are listed as: total length (femur, patella, tibia, metatarsus, tarsus). The number of spines is listed for each segment in the following order: prolateral, dorsal, retrolateral, ventral (in femora and patellae, ventral spines are absent, and the fourth digit is omitted in the spination formula). Abbreviations used in the text and figures are given below:

**ALE** anterior lateral eye;

**AME** anterior median eye;

**AW** anterior width of carapace;

**C** conductor;

**CH** clypeus height;

**dRTA** dorsal branch of RTA;

**E** embolus;

**EA** embolic apophysis;

**FD** fertilization duct;

**FE** femur;

**GA** glandular appendage;

**LL** lateral lobes;

**LS** lobal septum;

**MS** membranous sac;

**Mt** metatarsus;

**OL** opisthosoma length;

**OW** opisthosoma width;

**Pa** patella;

**PI** posterior incision of LL;

**PL** carapace length;

**PLE** posterior lateral eyes;

**PME** posterior median eyes;

**Pp** palp or palpus;

**PP** posterior part of spermathecae;

**PW** carapace width;

**RTA** retrolateral tibial apophysis;

**SP** spermophor;

**ST** subtegulum;

**T** tegulum;

**Ta** tarsus;

**Ti** tibia. I, II, III, IV – legs I to IV;

**vRTA** ventral branch of RTA;

**HUST** School of Nuclear Technology and Chemistry & Biology, Hubei University of Science and Technology, Xianning, Hubei, China (Y. Zhong).

## Taxonomy

**Family Sparassidae Bertkau, 1872**

**Subfamily Heteropodinae Thorell, 1873**

### Genus *Sinopoda* Jäger, 1999

#### Sinopoda yichangensis

Taxon classificationAnimaliaAraneaeSparassidae

Zhu, Zhong & Yang, 2020

CF374A5E-D80A-5A34-88EC-3853E2B61524

Figures 1 , 2 , 3

Sinopoda yichangensis Zhu, Zhong & Yang, 2020: 9, figs 4A–C, 5A–C, 6A, B (holotype male from Qiaoliao Village of Hubei Province, deposited in College of Life Science, Hubei University LJ-202001-ZY).

##### Material examined.

2♂, 3♀ (HUST 0002), Hubei Province, Yichang City, Wufeng County, Qiaoliao Village; 30.37°N, 110.54°E; alt. 986 m; 27.V. 2021, Yang Zhong leg.

**Figure 1.**
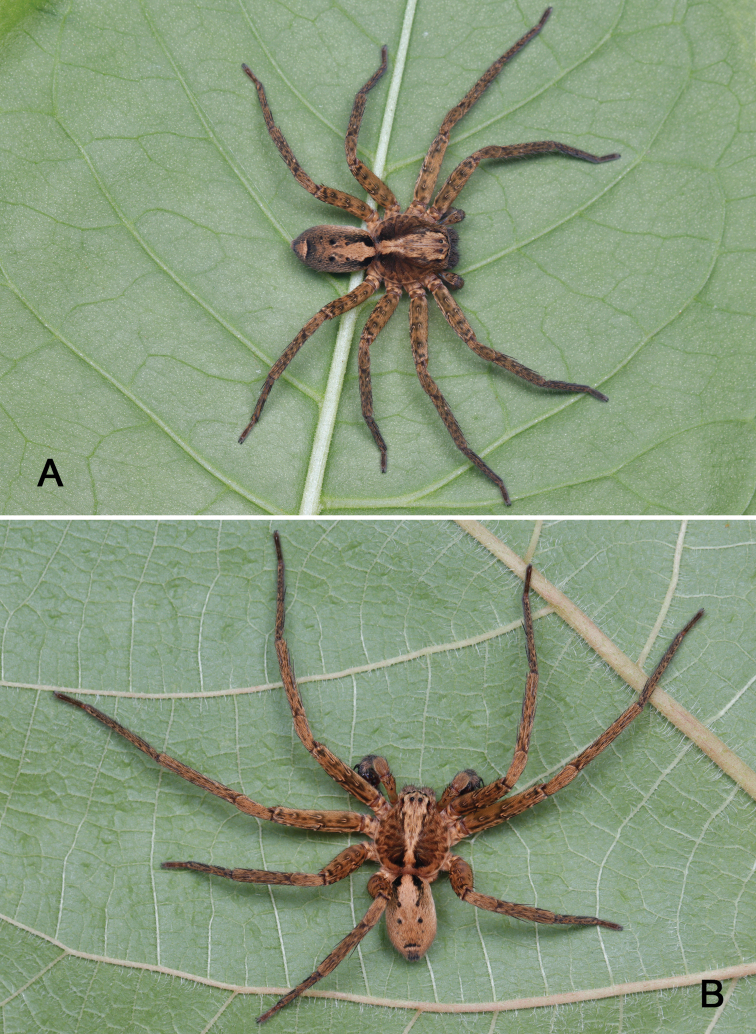
Photos of live *Sinopodayichangensis* Zhu, Zhong & Yang, 2020 **A** female **B** male.

##### Diagnosis.

**Figure 2.**
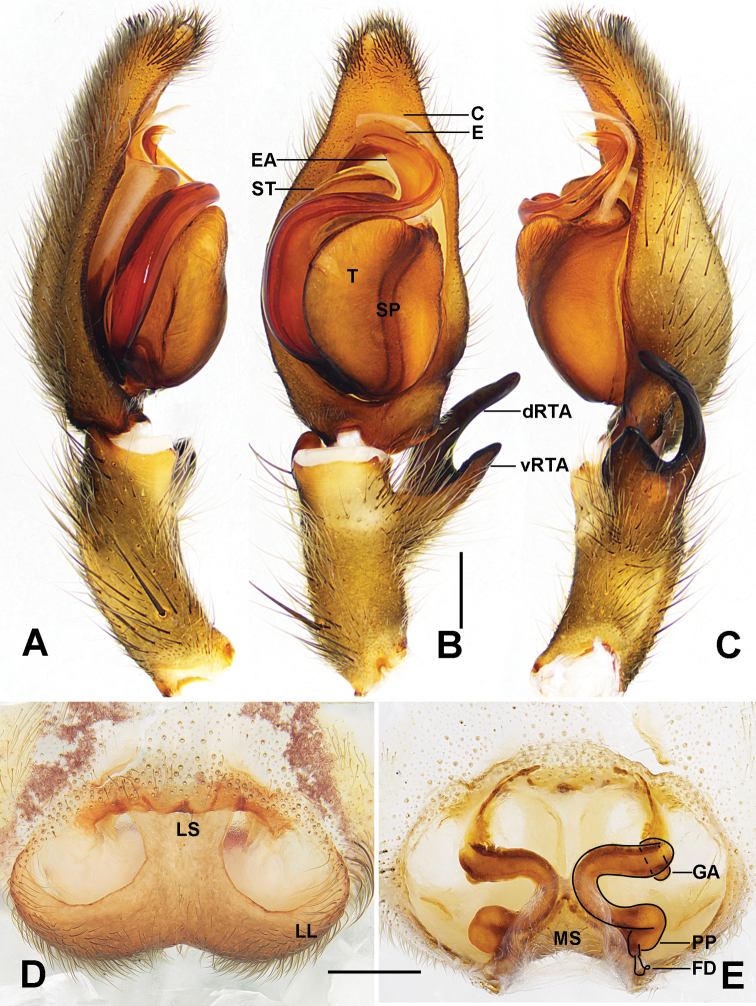
*Sinopodayichangensis* Zhu, Zhong & Yang, 2020 **A–C** left male palp (**A** prolateral view **B** ventral view **C** retrolateral view) **D** epigyne **E** vulva (**D** ventral view **E** dorsal view). Abbreviations: C – conductor, dRTA – dorsal retrolateral tibial apophysis, E – embolus, EA – embolic apophysis, FD – fertilization duct, GA – glandular appendage, LL – lateral lobes, LS – lobal septum, MS – membranous sac, PP – posterior part of spermathecae, SP – spermophor, ST – subtegulum, T – tegulum, vRTA – ventral retrolateral tibial apophysis. Scale bars: 0.5 mm.

This species resembles *Sinopodaangulata* Jäger, Gao & Fei, 2002 (Zhu et al. 2020: fig. 2A–E) by palp with an embolus distally filiform, as long as the embolic apophysis, and epigyne with lateral lobes fused without visible seam, and anterior part of the internal duct system not visible in dorsal view (Fig. 2E, see dotted line). They can be distinguished from the latter by the following characters: 1, embolus arising from tegulum at 7- to 8-o’clock position in ventral view (6-o’clock position in *S.angulata*); 2, tip of embolic apophysis with blunt end (pointed in *S.angulata*); 3, female epigyne with lobal septum ~ 1/3 of epigynal width (~ 1/4 in *S.angulata*); 4, female vulva with internal duct system not touching (touching along median line in *S.angulata*) (Fig. 2E).

##### Description.

**Male.** See Zhu et al. (2020).

##### Female.

**Figure 3.**
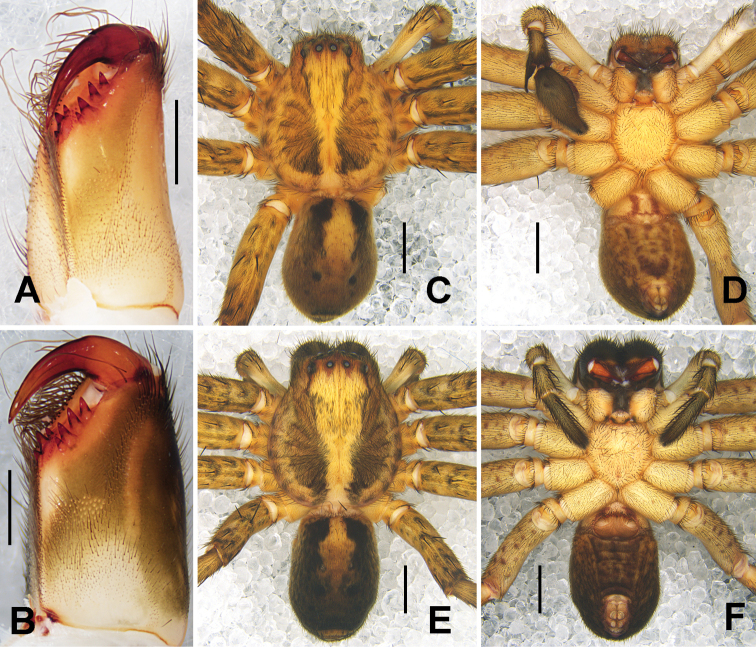
*Sinopodayichangensis* Zhu, Zhong & Yang, 2020 **A, B** cheliceral dentition, ventral view (**A** male **B** female) **C, D** male habitus (**C** dorsal view **D** ventral view) **E, F** female habitus (**E** dorsal view **F** ventral view). Scale bars: 0.5 mm (**A, B**); 2 mm (**C-F**).

PL 6.2, PW 5.4, AW 3.5, OL 5.0, OW 3.3. Eyes and interdistances: AME 0.20, ALE 0.34, PME 0.19, PLE 0.33, AME-AME 0.25, AME-ALE 0.15, PME-PME 0.51, PME-PLE 0.62, AME-PME 0.53, ALE-PLE 0.64, CHAME 0.25, CHALE 0.33. Spination: Palp: 131, 000, 2121, 1014; Fe: I–III 323, IV 321; Pa: I–IV 101; Ti: I 2026, III–IV 2226; Mt: I–II 1014, III–IV 3036. Measurements of palp and legs: Palp 7.1 (2.4, 1.4, 1.5, –, 1.8), I 16.9 (5.0, 2.6, 4.4, 3.4, 1.5), II 19.1 (5.6, 2.7, 4.9, 4.4, 1.5), III 16.2 (4.9, 2.4, 4.0, 3.5, 1.4), IV 17.4 (5.1, 2.0, 4.6, 4.2, 1.5). Leg formula: 2–4-1–3. Cheliceral furrow with three anterior and four posterior teeth, each furrow with 23 denticles (Fig. 3B). Copulatory organ as in diagnosis. Epigynal field wider than long, with short anterior muscle attachment bands, with one slit sensillum on each side close to the epigynal field. Glandular appendages short, extending only in anterior half of internal duct system. Internal duct system converging and strongly U-shaped. Fertilization ducts arising postero-laterally, curved. Membranous sac between fertilization ducts almost triangular (Fig. 2D, E). Coloration in ethanol: as in male, but slightly brighter (Fig. 3E, F).

##### Distribution.

Known only from the type locality.

##### Variation.

**Male (n = 2)**: Total length 13.5–16.5; prosoma 6.0–7.5 long, 5.0–6.2 wide, anterior width of prosoma 2.3–3.2; opisthosoma 7.5–9.0 long, 3.5–4.5 wide. Measurements leg I: total length 34.5–36.0, Fe 8.6–9.0, Pa 3.0–3.3, Ti 9.4–9.7, Mt 10.3–10.6, Ta 3.1–3.4. Spination: legs: Mt I–II 2024. **Female (n = 2)**: Total length 10.8–11.5; prosoma 5.5–6.5 long, 4.8–5.6 wide, anterior width of prosoma 3.0–3.8; opisthosoma 5.0–5.3 long, 2.8–4.0 wide. Measurements leg I: total length 15.6–18.0, Fe 4.8–5.3, Pa 2.4–2.8, Ti 4.3–4.7, Mt 3.0–3.6, Ta 1.3–1.6. Spination: legs: Ti I 2126, Mt I–II 2226.

## Supplementary Material

XML Treatment for Sinopoda yichangensis
